# Psychiatric implications of anti-seizure medications in epileptic population

**DOI:** 10.1186/s12883-024-03670-8

**Published:** 2024-05-21

**Authors:** Bushra Khalid, Zaid Waqar, Soban Khan, Ijaz Ali, Naheed Afzal, Anum Irfan, Waleed Malik, Malik Muhammad Adil, Amina Saddiqa, Maryam Khalil, Zeeshan Munawar

**Affiliations:** Pakistan Institute of Medical sciences, Islamabad, Pakistan

**Keywords:** Epilepsy, Antiseizure medications, Psychiatric comorbidities, Valproate, MRI findings

## Abstract

**Background and objective:**

Epilepsy is a prevalent neurological disorder that affects a significant number of individuals globally. This condition is associated with a high occurrence of psychiatric comorbidities, which can significantly affect the quality of life of individuals affected. The aim of this study was to investigate the association between antiseizure therapies and the likelihood of psychiatric comorbidities in individuals with epilepsy.

**Methodology:**

Data for this study was gathered from the Neurology referral center in Islamabad, Pakistan. A standardized questionnaire was utilized to gather data from 120 individuals diagnosed with epilepsy. The survey consisted of inquiries regarding the management of seizures, the utilization of anti-seizure medications, and the presence of psychiatric comorbidities. The data was analyzed using the Statistical Package for the Social Sciences (SPSS).

**Results:**

The findings indicated that individuals who were using multiple antiseizure medications had a notably higher likelihood of having psychiatric comorbidities in comparison to those who were on mono therapy (*p* = 0.010). suggests that patients with unsuccessful seizure control are more probable to have psychiatric comorbidities as compared to those with good seizure control (*p* = 0.029).

**Conclusion:**

To conclude poor seizure control and poly therapy are associated with increased risk of psychiatric comorbidities.

## Introduction

An epileptic seizure is a temporary event characterized by aberrant and excessive neuronal activity in the brain, resulting in the manifestation of signs and/or symptoms [1]. Seizures can be classified as either focal, which means they originate in networks limited to one hemisphere of the brain, or generalized, which means they arise within and rapidly engage networks in both hemispheres [2]. Epilepsy is a neurological ailment that is characterized by a long-lasting tendency to experience epileptic seizures. It also has various effects on the brain’s biology, cognition, psychology, and social aspects [3]. The clinical definition of epilepsy, according to the International League Against Epilepsy, necessitates either two unprovoked seizures occurring more than 24 h apart, one unprovoked seizure with a future 10-year risk of recurrence equivalent to that of an individual with epilepsy (60% risk, determined by clinical history, physical examination, and diagnostic tests), or the diagnosis of an epilepsy syndrome [4]. 

Epilepsy is a prevalent disorder that affects individuals of various ages, genders, and socioeconomic backgrounds. The Global Burden of Disease Study 2013 Collaborators found that it is the third most prevalent neurological cause of years lived with disability worldwide [5]. 

Psychiatric issues frequently occur in individuals diagnosed with epilepsy. To fully comprehend the influence of psychiatric diseases on individuals with epilepsy, it is crucial to first grasp the idea of ‘comorbidity’. A comorbidity can be defined as a medical condition that either occurs at the time of diagnosis or develops over the course of the disease, but is not caused by the disease itself [6]. 

From an epidemiological standpoint, it is expected that psychiatric comorbidities in epilepsy would co-occur with the disease at some point throughout its normal progression, but they should not arise as a direct consequence of the condition. Common pathways may contribute to the development of both epilepsy and a psychiatric disorder in the same individual. Despite limited data, there is evidence to suggest that individuals with focal epilepsies originating from the temporal (with a risk of approximately 60%) or extratemporal (with a risk of approximately 54%) regions may have a higher likelihood of experiencing a psychiatric comorbidity compared to those with a generalized epilepsy syndrome (with a risk of approximately 37%). This indicates the presence of distinct pathophysiological mechanisms [7]. 

Given this context, it is crucial to consistently utilize this definition of comorbidities in the context of epilepsy. Psychiatric disorders can manifest either as temporary disturbances directly caused by a seizure (referred to as ‘periictal’ or ‘postictal’ psychiatric disturbances) or as independent conditions occurring alongside epilepsy (known as ‘interictal’ psychiatric disorders). For the purposes of this paper, we will define comorbidity as the phenomenon we just mentioned. Although peri- and postictal psychiatric disorders are prevalent and have a significant impact on individuals with epilepsy, particularly those with drug-resistant epilepsy, this article will primarily focus on interictal psychiatric conditions that coexist with epilepsy.

Anticonvulsant medications are the primary treatment for epilepsy. However, new research has indicated that these treatments may have psychological side effects in individuals with epilepsy. Valproate, a frequently used medication for seizures, has been associated with a heightened likelihood of experiencing low mood and anxiety [8]. In addition, it has been found that Levetiracetam, a commonly prescribed antiseizure medication, is associated with higher incidences of aberrant behavior, aggression, and mood disorders in individuals with epilepsy [9].

To fully understand the potential psychiatric side effects of antiseizure therapies, it is crucial to investigate how often and in what manner individuals with epilepsy who are using these medications also experience psychiatric conditions. The objective of this study is to determine the prevalence of psychiatric comorbidities among individuals with epilepsy who are using antiseizure medications, and to compare the prevalence of psychiatric comorbidities between individuals who are using single medications and those who are on polytherapy.

## Methodology

### Study design

cross-sectional study conducted in a hospital in Islamabad, Pakistan. The study was approved by the official review board of Department of neurology.

### Objective

The objective of the study was to investigate the prevalence of psychiatric comorbidities in individuals with epilepsy who are undergoing treatment with antiseizure medications, and to compare the prevalence of psychiatric comorbidities between individuals taking polytherapy and monotherapy.

The study also investigated the potential correlation between distinct categories of antiseizure medications and the occurrence of psychiatric comorbidities in individuals with epilepsy.

### Sample size

Sample Size was calculated using WHO (world health organization) sample size calculator.

Margin of error: 9%.

Confidence Interval: 95%.

Response Distribution: 50%.

Sample Size: 118 (Author decided to include round number 120 patients).

### Study population

The study comprised 120 adult individuals (age > 18 years) who had been diagnosed with epilepsy and were undergoing treatment with antiseizure medications. Patients with a preexisting psychiatric history prior to the diagnosis of epilepsy were not included in the study.

### Data collection

Information was gathered from medical records and interviews with individuals who were affected, utilizing a standardized questionnaire. This study utilized a questionnaire that encompassed inquiries regarding demographic factors (age, gender, education, and profession), attributes of the complaints (seizure type, duration, and MRI brain findings), history of antiseizure medication usage (type and duration of treatment), seizure control, and presence of psychiatric comorbidity. All the patients were screened for psychiatric co-morbidity by certified psychiatrist. Hamilton anxiety and depression scores (HAM-A, HAM-D) were used for scoring of anxiety and depression. DSM-V Inventories for personality disorders and psychoses were used. Since the above mentioned scored are already validated internationally they were selected by the authors.

### Data analysis

Descriptive statistics were employed to summarize the demographic and medical characteristics of the study population. The distribution of antiseizure medicines and mental comorbidities within the study population was analyzed using frequency statistics and frequency tablesThe connection between several parameters, such as seizure control or MRI results, and the occurrence of psychiatric comorbidities in individuals receiving antiseizure medicines was examined using cross-tabulation and odds ratio calculation.

### Statistical analysis

All of the statistical analyses were executed through SPSS version 25.

## Results

The study population consisted of 120 patients with a mean age of 32.98 years (standard deviation = 13.24). The age at which epilepsy first appeared varied from 1 to 59 years, with an average age of onset of 25.26 years (standard deviation = 15.89). Out of the 120 patients, the majority (60%) endured generalized seizures, while 24.2% had focal seizures, and 10% had secondary generalized seizures. Only a small number of patients (5.8%) had seizures that weren’t specified.

Among the study population, the prevalence of psychiatric comorbidities was 47.5%. In these 47.5% patients, depression was being the most prevalent at 31.7% 15.8% had anxiety 5% had psychotic symptoms. Low IQ was found in 9.2% while personality disorders and PNES were seen in 5.8% and 6.7% respectively. MRI of the brain was reported normal in 42.5% patients 9.2% had abnormalities in frontal lobe, 11.7% had temporal lobe abnormalities apart from hippocampal sclerosis, while5.8% had hippocampal sclerosis on MRI of the brain. Parietal and occipital lesions were seen in 18.3% and 4.2% respectively. 8.3% had space occupying lesions on MRI brain. Out of the 120 cases, 97(80.8%) reported effective seizure control while 23(19.2%) reported ineffective control. Table 1 shows the results of seizure control among the study patients.

**Table 1:** Seizure control among study participants

Seizure control					
		Frequency	Percent	Valid Percent	Cumulative Percent
Valid	Effective	97	80.8	80.8	80.8
	Ineffective	23	19.2	19.2	100.0
	Total	120	100.0	100.0	

The prevalence of psychiatric comorbidities was higher among individuals taking multiple antiseizure medicines compared to those who are on monotherapy odds ratio:1.352 (95% CI 1.129–1.619) (p value 0.010) as shown in Table 2. There was no statistically significant relationship in the occurrence of psychiatric comorbidities in individuals on Valproate and those taking Levetiracetam the two most commonly prescribed anti-epileptic drugs. Presence of abnormalities on neuroimaging had a statistically non-significant effect on development of psychiatric morbidity (p-value 0.233).

**Table 2:** Crosstabs of Anti-epileptic Drug Poly Therapy- with Presence of psychiatric comorbidity

AED Polytherapy * Psychiatric comorbidity Crosstabulation					
			Psychiatric comorbidity		Total
			Yes	No	
AED PolyTherapy	Yes	Count	31	3	34
		% within AED PolyTherapy	91.2%	8.8%	100.0%
	No	Count	58	28	86
		% within AED PolyTherapy	67.4%	32.6%	100.0%
Total		Count	89	31	120
		% within AED PolyTherapy	74.2%	25.8%	100.0%
		**Odds Ratio**	1.352	95% Confidence Interval (1.129–1.619)	
		**P-value**	0.010		

The Table 3, is indicating a significant relationship between seizure control and psychiatric comorbidity ( *p* = 0.029). The data suggests that patients with unsuccessful seizure control are more probable to have psychiatric comorbidities as compared to those with good seizure control.

**Table 3:** Crosstabs of Seizure control - with Presence of psychiatric comorbidity

Seizure control * Psychiatric comorbidity Crosstabulation					
			Psychiatric comorbidity		Total
			Yes	No	
Seizure control	Effective	Count	67	30	97
		% within Seizure control	69.1%	30.9%	100.0%
	Ineffective	Count	22	1	23
		% within Seizure control	95.7%	4.3%	100.0%
Total		Count	89	31	120
		% within Seizure control	74.2%	25.8%	100.0%
		**Odds Ratio**	9.851	95% Confidence Interval (1.26–76.50)	
		**P-Value**	0.029		

## Discussion

This study aimed to investigate the correlation between the utilization of antiepileptic medicines and the likelihood of developing psychiatric comorbidities in individuals with epilepsy. The findings of this study provide evidence that epileptic patients who are on several antiepileptic drugs and those with inadequate seizure control are more susceptible to psychiatric comorbidities compared to those who are not using these medications. This discovery aligns with prior research that has documented a greater occurrence of psychiatric comorbidities in individuals with epilepsy who have in effective seizure control [10, 11], and a higher occurrence of psychiatric comorbidities in individuals with epilepsy who are using numerous antiepileptic medications [12, 13]. .

The current investigation established that the predominant psychiatric comorbidity observed in individuals with epilepsy was anxiety, followed by depression. These findings align with previous research that have documented a significant prevalence of anxiety and sadness in individuals with epilepsy [14, 15]. .

The study also investigated the correlation between certain antiepileptic medicines and the likelihood of psychiatric comorbidities.

The findings indicate that individuals who were prescribed valproate or levetiracetam no were more prone to have psychiatric comorbidities compared to those who were not prescribed valproate. This conclusion is not consistent with other research that have found an increased risk of psychiatric comorbidities in those using valproate [16, 17]. .

This study not only discovered a higher likelihood of psychiatric comorbidities in individuals with epilepsy who are using antiseizure medications, but it also revealed some favorable outcomes. Initially, the study established that a significant majority of individuals (80.8%) reported successful management of seizures with their existing treatment regimen. This finding is significant since controlling seizures is a crucial element of epilepsy management and can considerably improve the quality of life for those with epilepsy [18]. 

Moreover, a significant proportion of patients had normal MRI brain scans, indicating a favorable prognosis and implying a reduced likelihood of encountering issues related to structural anomalies in the brain [19]. 

To conclude poor seizure control and poly therapy are associated with increased risk of psychiatric comorbidities. These maybe related to underlying severity of biologic disease process or the result of multiple drug adverse effects and drug interactions, for which purposes further studies are needed to elaborate on that fact.

### Conclusion

With epilepsy, psychiatric comorbidities are frequent. The correlation between epilepsy and a wide range of psychiatric disorders may be explained by common pathophysiological mechanisms. It is imperative that these circumstances be identified, and future studies should investigate whether particular psychiatric symptoms are particularly associated with the neuroanatomical mechanisms determining the kind of epilepsy (e.g., temporal versus extratemporal, or generalized versus localized). Associated psychiatric comorbidities are detrimental to quality of life, seem to predict worse outcomes from seizures, and may be linked to non-adherence to medicine. Future research should therefore concentrate on accurately identifying these disorders because their symptoms may not be the same as those of those who do not have epilepsy.

## Data Availability

SPSS data sheets available on reasonable request.

## Abbreviations

MRI: Magnetic Resonance Imaging
